# Pluripotency Factors on Their Lineage Move

**DOI:** 10.1155/2016/6838253

**Published:** 2015-12-06

**Authors:** Clair E. Weidgang, Thomas Seufferlein, Alexander Kleger, Martin Mueller

**Affiliations:** ^1^Department of Internal Medicine I, Ulm University Hospital, 89069 Ulm, Germany; ^2^Department of Anesthesiology, Ulm University Hospital, 89069 Ulm, Germany

## Abstract

Pluripotent stem cells are characterised by continuous self-renewal while maintaining the potential to differentiate into cells of all three germ layers. Regulatory networks of maintaining pluripotency have been described in great detail and, similarly, there is great knowledge on key players that regulate their differentiation. Interestingly, pluripotency has various shades with distinct developmental potential, an observation that coined the term of a ground state of pluripotency. A precise interplay of signalling axes regulates ground state conditions and acts in concert with a combination of key transcription factors. The balance between these transcription factors greatly influences the integrity of the pluripotency network and latest research suggests that minute changes in their expression can strengthen but also collapse the network. Moreover, recent studies reveal different facets of these core factors in balancing a controlled and directed exit from pluripotency. Thereby, subsets of pluripotency-maintaining factors have been shown to adopt new roles during lineage specification and have been globally defined towards neuroectodermal and mesendodermal sets of embryonic stem cell genes. However, detailed underlying insights into how these transcription factors orchestrate cell fate decisions remain largely elusive. Our group and others unravelled complex interactions in the regulation of this controlled exit. Herein, we summarise recent findings and discuss the potential mechanisms involved.

## 1. Introduction

Pluripotency represents three essential features: first the capacity of indefinite self-renewal, second the ability of giving rise to differentiated progeny of nearly all lineages of the mature organism, and last the generation of chimeric embryos upon injection into the inner cell mass (ICM) of a blastocyst [1]. The complexity of the regulatory networks, which maintain pluripotency, has previously been described [2]. Complex interactions between signalling axes precisely regulate various states of pluripotency, such as the ground and the primed state, and act in concert with a combination of key transcription factors (TFs). Vice versa, we have gained a great body of knowledge on key players guiding pluripotent stem cells (PSCs) towards differentiation [3, 4]. In line, regulatory networks both in PSCs and in the developing organism are tightly balanced as small changes can break down the entire pluripotency network leading to differentiation [3]. In recent years, studies have revealed novel facets of these core factors balancing a controlled exit of pluripotency and guiding early steps of differentiation [5]. Herein, subsets of pluripotency-maintaining factors have been shown to adopt new roles during lineage specification and have been grouped into neuroectodermal and mesendodermal sets of embryonic stem cell genes [5]. Accordingly, for example, Nanog, Tbx3, Klf5, and Oct3/4 regulate the exit towards mesendoderm while Sox2 regulates differentiation towards a neuroectodermal fate [5]. However, detailed underlying mechanisms of how these TFs orchestrate cell fate decisions remain largely elusive. Aiming to close this gap of knowledge, we have recently studied the mechanism of Tbx3 to regulate mesendodermal fate. Briefly, we have defined novel facets of Tbx3 which directly activates core regulators of the mesendodermal lineage and indirectly induces differentiation via a paracrine Nodal signalling loop [6]. Thus, our data illustrate the dual complexity of pluripotency TFs to gate fate determination.

In turn, the current review will at first give a brief insight into essential signalling pathways and TFs maintaining the self-renewal state. Secondly, we will summarise recent findings of pluripotency-associated factors that have an important impact on early lineage specification processes.

## 2. ESC Pluripotency and Its Signalling Pathways

Most of the knowledge on pluripotency has been obtained using mouse embryonic stem cells (mESCs) as a research tool [8–11].* In vitro* pluripotency has various shades mirroring distinct* in vivo* counterparts, an observation which has led to the definition of two pluripotency states: naïve and primed [12–14]. Herein, the early ICM of the blastocyst requires a maturation step to obtain clonal pluripotent colonies, while E4 and E4.5 preimplantation epiblast cells from the blastocyst robustly give rise to naïve pluripotent cells capable of single-cell culture [15]. To capture naïve pluripotency* in vitro*, cells isolated from the ICM are cultured serum-free in the presence of leukaemia inhibitory factor (LIF) and bone morphogenetic protein (BMP) or under LIF/2i-culture conditions (outlined below) [16, 17]. In contrast, cells derived from the postimplantation epiblast are not any longer single-cell culture permissive and require distinct signalling input such as FGF- (fibroblast growth factor-) supplementation to remain within the pluripotent state. These cells are* in vitro* referred to as epiblast stem cells (EpiSCs). They are characterised by primed pluripotency and exhibit a slightly reduced differential potential, thus surrogating a more advanced pregastrulation stage [18, 19]. A large body of knowledge has been gained in the past years, deciphering signalling axes with an impact on the pluripotent state. Basically, the mitogen-activated protein kinase (MAPK) signalling pathway and glycogen synthase kinase 3 (GSK3) signalling pathway negatively influence pluripotency, while LIF/STAT3 (signal transducer and activator of transcription 3) and BMP/SMAD (mothers against decapentaplegic) signalling are complementary beneficial [16, 17].

### 2.1. LIF/STAT3 Signalling

**Figure 1:**
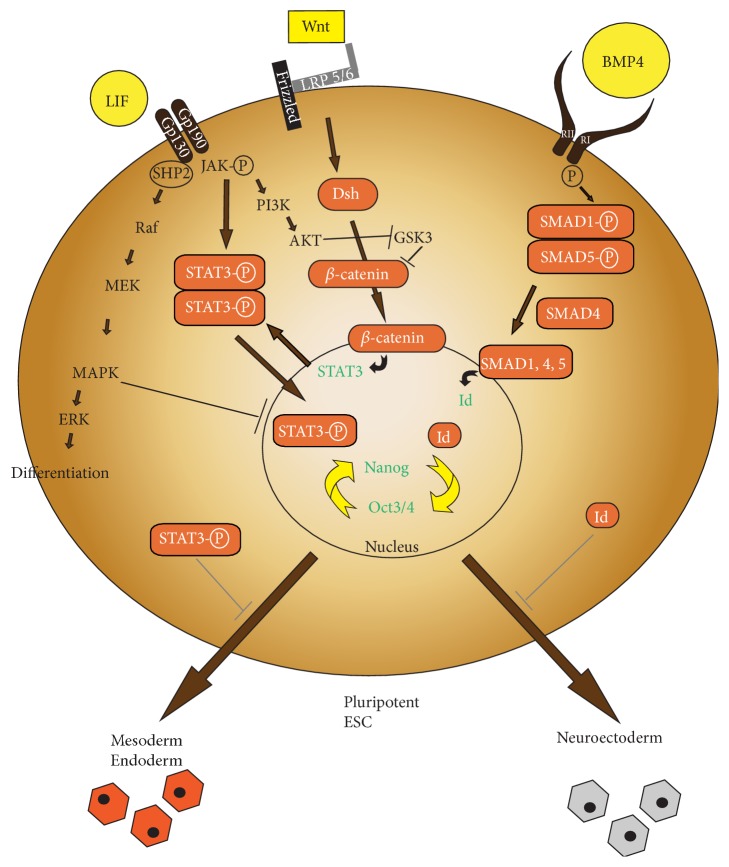
Signalling pathways regulating pluripotency in mESCs. A schematic representation of the main extrinsic pathways (LIF, BMP, and WNT) regulating pluripotency in mESCs. Upon LIF binding to its receptor, Gp130 phosphorylates JAK, which in turn phosphorylates STAT3. Phosphorylated STAT3 operates as a transcription factor which sustains the pluripotency state in mESCs and further inhibits endodermal and mesodermal differentiation. LIF further inhibits GSK3 via PI3K. GSK3 stabilises the self-renewal state by reducing the ubiquitin dependent degradation of *β*-catenin. WNT proteins bind the Frizzled receptor which in turn forms a complex with LRP5/6 protein and signal downstream via Dsh and *β*-catenin. *β*-catenin accumulates in the cytoplasm and nucleus resulting in STAT3 transcription. STAT3 is activated through JAK phosphorylation. Upon BMP4-receptor complex formation, BMP RI phosphorylates SMAD1 and SMAD5 which then interacts with SMAD4 resulting in Id gene transcription and maintenance of the pluripotency state. Image modified after Hao [7]. Dsh: dishevelled, ERK: extracellular receptor kinase, Id: inhibitor of differentiation, JAK: Janus kinase, and mESCs: murine embryoid stem cells. LIF: leukaemia inhibitory factor, MAPK: mitogen-activated protein kinase, Oct3/4: octamer binding transcription factor 3/4, PI3K: phosphatidylinositide 3′-kinase, SMAD1/4/5: mothers against decapentaplegic homolog 1/4/5, STAT3: signal transducers and activators of transcription 3, RI: receptor type I, RII: receptor type II, and WNT: wingless-related MMTV integration site family.

mESCs are commonly retained in the pluripotent state by culturing them on a feeder layer composed of mouse inactivated fibroblasts (MEFs) in the presence of the cytokine LIF [20–22]. LIF functions via a complex signalling axis and finally activates core TFs, such as octamer binding transcription factor 3/4 (Oct3/4), sex determining region Y- (SRY-) box  2 (Sox2), and Nanog, which orchestrate the self-renewal state [23, 24].* In vivo*, their expression levels overlap both in the ICM and in the epiblast [24], hence being able to interact and activate pluripotency-associated genes, while repressing lineage specific differentiation programmes. LIF signals via three different pathways. However, its key effect in sustaining the pluripotent state is implemented by activating the TF STAT3 via phosphorylation [25]. STAT3, the crucial downstream target of LIF, directly binds the distal enhancers of Oct3/4 and Nanog [26] and further pluripotency TFs [27] such as Krüppel-like factor 4 (as Klf4) [28]. Thereby it modulates their expression levels for propagation of the murine pluripotent phenotype. In the absence of LIF signalling, mESCs differentiate to the mesodermal and endodermal lineage [29]. Therefore, LIF limits mESC differentiation towards mesendoderm in favour of maintaining the pluripotent state in a closely regulated balance with BMP signalling [16]. Despite its essential role in self-renewal* in vivo* and* in vitro*, LIF also participates in regulating a differentiation programme driven by the extracellular-signal-regulated kinase (ERK). The ERK signalling cascade promotes early differentiation* in vitro* and* in vivo* [30, 31]. Thus, LIF seems to modulate stem cell fate between self-renewal and lineage specification by regulating expression levels of STAT3 and ERK [32]. In summary, LIF represents a critical component in maintaining the self-renewal state in mESC culture by activating pluripotency-associated TFs. Of note, LIF-dependence can be overcome at least temporarily by chemical inhibition of MAPK and GSK3 signalling (“2i”) but still remains beneficial, an observation establishing LIF-2i as the gold standard of serum-free mESC culture (Figure 1) [15, 33, 34].

### 2.2. BMP Signalling

In a serum-free cell culture LIF is solely not able to maintain mESC pluripotency [16]. BMP4 is a secreted signalling molecule belonging to the transforming growth factor beta (TGF*β*) family and represents an attested ectoderm-antagonist [16, 35, 36]. BMP4 (and BMP2) successfully replaces serum requirements resulting in propagation of pluripotency and inhibition of multilineage differentiation in the presence of LIF. In the absence of LIF, BMP promotes mesodermal differentiation [37] at the expense of the neural lineage [38] (Figure 1). Further, the BMP pathway also has the potential to promote differentiation of mESCs into the trophoblast lineage via caudal type homeobox  2 (Cdx2) when cultured under defined conditions [39].

In the preimplantation embryo, BMP4 becomes induced in the ICM of the early blastocyst while its expression peaks in the epiblast of the E4.5 blastocyst and decreases afterwards. This decrease of BMP signalling in the postimplantation epiblast coincides with the upregulation of Nodal/Activin signalling and subsequent target gene expression such as Lefty1 and Lefty2 pointing at opposing roles of Nodal and BMP signals at various developmental stages [15, 40–42]. This* in vivo* observation matches the requirement of low-dose Nodal/Activin signalling to maintain the primed state of pluripotency [43]. The opposing effects of Nodal and BMP signalling are obviously relevant not only for lineage commitment but also for fine tuning BMP signalling in the naïve state involving an intracellular mechanism via SMAD7 to modulate SMAD1/SMAD5 levels [4, 40, 44]. In summary, LIF is dependent on BMP signalling in a serum-free mESC culture, while both signalling axes are strongly dependent on a precise dosage regulation as any imbalance between both pathways drives exit from pluripotency to various fates. In the absence of LIF, BMP signalling directs mESCs towards the mesendoderm and trophoblast lineage [45, 46].

### 2.3. Small Molecules Capture Ground State Pluripotency

Previously, the Smith Laboratory identified two small molecules, PD0325901 (PD03) and CHIR99021 (CHIR), which can substitute for LIF and BMP under defined culture conditions to facilitate ground state pluripotency in mESC (2i conditions) [17]. PD03 inhibits MEK, a downstream target of FGF signalling, important for trophectodermal lineage differentiation in early embryos [47]. Further, mESC pluripotency and particularly viability is promoted by increased WNT (wingless-type MMTV integration site family) signalling via CHIR, a specific GSK3 inhibitor [17, 48]. Nuclear expression level of *β*-catenin, the core effector of WNT signalling [49], is regulated by a multiprotein destruction complex [50]. Herein, GSK3 inhibition stabilises the self-renewal state by diminishing ubiquitin dependent degradation of *β*-catenin and repressing Tcf3, a known transcriptional repressor of target genes [34]. Tcf3 cooccupies and represses Oct3/4, Sox2, Nanog, and estrogen-related receptor *β* (Esrrb) [51–54], suggesting its role as a crucial regulator of the transcriptional control of pluripotency in mESCs. Regarding WNT signalling and its impact on mESC pluripotency, *β*-catenin interacts with Oct3/4 [34, 55] and Sox2 [56, 57] and is activated by Nanog via Dickkopf-related protein 1 (Dkk1) repression [58] to reinforce the self-renewal state. Despite its role in maintaining pluripotency, canonical WNT/*β*-catenin signalling participates in body axis patterning, primitive streak and extraembryonic lineage formation, and mesoderm specification through Brachyury [59–63]. In summary, inhibition of MAPK and GSK3 signalling together with LIF-supplementation under serum-free culture conditions is capable of capturing naïve pluripotency robustly from various preimplantation stages and can preserve ground state conditions with erased lineage fates [15, 33].

## 3. Lineage Specific Classification of Pluripotency Transcription Factors

Despite acting during mESC self-renewal, pluripotency-associated factors also connect the transition from pluripotency towards lineage specification [4, 5]. The pluripotent state is maintained by a unique network of directly interacting TFs (including Oct3/4, Sox2, Nanog, Tbx3, and Klf4/5). These TFs either inhibit target gene expression levels required for lineage differentiation or sustain the expression of one another [64–66]. However, upon differentiation, controlled mesendodermal and neuroectodermal commitment requires the reorganisation of the circuit to allow onset of lineage specific programmes. Herein, extracellular clues, such as WNT, BMP, and TGF*β*, further impact and regulate cell fate choice [67]. Interestingly, certain pluripotency factors are not simply downregulated but instead their expression is either sustained or even upregulated for a short time window during pluripotency exit [4, 5]. This does not occur randomly but instead is lineage and factor specific: Basically, we can distinguish three groups: mesendoderm-class genes (e.g., Kf4/5, Nanog, Oct3/4, and Tbx3), neuroectoderm-class genes (e.g., Sox2), and extraembryonic-class genes (e.g., spalt-like transcription factor 4 (Sall4)) [5]. Mechanistically, TFs bind asymmetrically in regulatory regions on the one hand promoting the respective lineage but on the other hand repressing the other, thus ensuring tightly regulated cell fate choices. In the following sections, we aim to discuss pluripotency factors, which have recently been shown to have such a dual function, namely, pluripotency and early cell fate choice [68].

**Figure 2:**
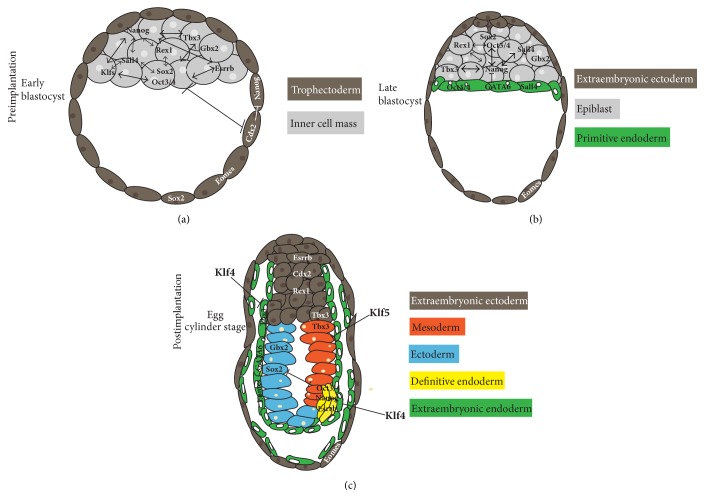
Pluripotency factor expression during early embryonic development. Pluripotency factors are expressed throughout the pluripotency state and subsequently govern early lineage decisions in mouse. (a) The early blastocyst consists of the ICM and the outer trophectoderm. Pluripotency factors are expressed in the ICM, however, interacting with trophectodermal genes for proper lineage development. (b) At the late blastocyst stage, the ICM segregates into the epiblast, notably exhibiting slightly different gene expression patterns, and into the extraembryonic endoderm, later giving rise to yolk sac. Further, the trophectoderm has formed the extraembryonic ectoderm lineage which will generate the placenta. (c) After implantation, the primitive ectoderm, which has origins in the epiblast, forms the four embryo-derived stem cell lineages (ectoderm, mesoderm, endoderm, and germ lineage). Notably, pluripotency factors guide early embryonic decision according to their expression profile, respectively. ICM: inner cell mass.

However, before continuing we want to briefly summarise the earliest embryonic cell fate decisions for better comprehension (Figure 2). The zygote represents the earliest stage of the early mammalian embryo. From here, the cells undergo several series of cleavage divisions producing the morula [69]. Subsequently, the first cell decision occurs during the transition from morula to blastocyst by differentiating into two distinct lineages. The outer cell layer of the blastocyst forms the trophectoderm, whereas the inner cells develop to the ICM [70]. The trophectoderm proliferates further into the extraembryonic ectoderm and trophoblast, later giving rise to the placenta. Prior to implantation the ICM undergoes the second cell fate decision by differentiating into either the epiblast (later forming the primitive ectoderm and subsequently giving rise to the three germ layers among others) or the primitive endoderm [69]. The primitive endoderm cells form both visceral and parietal endoderm and finally give rise to the yolk sac [71]. Prior to mouse gastrulation, the initially symmetrical embryo is prepatterned by regional distinctions in gene expression profiles and levels of signalling pathways along the embryonic axes [4]. The formation of the primitive streak as the first obvious sign of germ layer formation is driven by gradients of growth factor signalling such as Nodal and canonical WNT at the posterior pole of the embryo, accompanied by the expression of early differentiation marker genes [4, 40]. The regulatory events that define the timing and site of gastrulation initiation still remain rather unclear. The formation of a transient precursor cell population located in the region of the anterior primitive streak reflects one of the earliest events during gastrulation. Definitive endoderm and anterior mesoderm derivatives, including cardiovascular and head mesenchyme progenitors, originate from these precursors. Notably, this cell population, referred to as mesendoderm, is marked by the expression of marker genes such as Eomesodermin (Eomes), Forkhead-Box-Protein A2 (Foxa2), Chordin (Chrd), Goosecoid (Gsc), and LIM-homeobox  1 (Lhx1) [72].

### 3.1. Mesendodermal-Class Genes

#### 3.1.1. Octamer Binding Transcription Factor 3/4 (Oct3/4): Indispensable for Lineage and Pluripotency

Oct3/4 has gained much attention as the key regulator of mESC pluripotency* in vitro* and* in vivo* [73]. Oct3/4 belongs to the POU family and, as a master regulator of pluripotency, it functions in a complex, consisting of Nanog, Oct3/4, and Sox2 [74]. Hereby, it crucially balances gene expression levels of the pluripotency circuitry [75]. STAT3 is able to directly bind and regulate Oct3/4 to maintain self-renewal [26]. Also, both have been shown to activate similar target genes [3]. Previous work describes Oct3/4 as a gatekeeper both for maintaining self-renewal and also in modulating stem cell fate choice in a dose-dependent manner [76]. In this respect, the Chambers Laboratory demonstrated that low Oct3/4 levels, as seen in heterozygous mESCs, were sufficient to maintain mESC pluripotency [77]. This was especially due to elevated promoter binding of Oct3/4 to pluripotency-associated factors (e.g., Esrrb, Klf4, Nanog, and Tbx3) and increased WNT signalling and LIF sensitivity. In contrast, elevated Oct3/4 levels destabilised the pluripotency network resulting in FGF-dependent differentiation [77]. In this sense, alternate partners of Oct3/4 can define the switch between pluripotency and lineage commitment: while in the pluripotent state Oct3/4 sustains Sox2 expression, the switch to activate Sox17 instead marks a critical event during mesendodermal differentiation. This process is triggered not only by a change in target gene expression but in a noncell autonomous manner by the secretion of paracrine factors further favouring mesendodermal differentiation [78, 79]. Moreover, Hogan et al. analysed the impact of Oct3/4 on regulating mESC differentiation based on previous knowledge that mESCs differentiation is the result of an interplay between transcriptional regulation and chromatin organisation [80, 81]. In this regard, exit from pluripotency was facilitated through intermittent homologous pairing of the Oct3/4 allele. This was mediated by a locus initially described as the Oct/Sox-binding element within the Oct3/4 promoter region. Besides, Oct3/4 is also known to undergo alternative promoter binding at the stage when loss of pluripotency occurs towards differentiation [82].

In the embryo, Oct3/4 maintains the ICM and guides proper trophectoderm segregation upon differentiation. As Cdx2 expression promotes the trophectoderm lineage [83], it is not surprising that Oct3/4 was identified as a negative regulator of Cdx2 [84]. The Oct3/4-Cdx2 complex (possibly together with Sall4 as mentioned below) specifies lineage formation in the early embryos by reciprocal inhibition* in vivo* and* in vitro* [85] (Figure 2).* In vivo*, Oct3/4 guides mesendodermal differentiation and further suppresses neuroectodermal gene expression programmes [5, 86]. Preceding mouse development, Oct3/4 is expressed in the primitive endoderm [87] (Figure 2). Conditional deletion of Oct3/4* in vitro* promotes mESCs to commit towards the trophectoderm lineage via Cdx2 and Eomes [3, 73, 84, 88]. Oct3/4-deficient mouse embryos develop to the blastocyst stage but fail to form a consistent pluripotent ICM resulting in embryonic lethality due to differentiation into the extraembryonic trophoblast lineage [73]. Thus, the acquisition of ICM identity is supposed to be dependent on Oct3/4 functions [84]. Indeed, a recent study revealed a transient ICM formation in Oct3/4-deficient embryos, due to elevated Nanog expression levels [89]. Moreover, these embryos were lacking a functional primitive endoderm; however, this was rescued by stage specific supplementation of FGF4 [89]. This study was able to reveal a critical role of Oct3/4 facilitating early lineage decisions in ICM cells towards either epiblast or primitive endoderm by attenuating Nanog expression levels and further promoting primitive endoderm formation in an FGF-dependent manner [89]. However, due to its importance in governing lineage decisions in the early blastocyst, it is not possible to date to isolate mESCs from Oct3/4-deficient embryos [73].

Taken together, Oct3/4 belongs to key pluripotency TFs, which maintains mESC pluripotency. Its tightly regulated expression levels drive proper mESC differentiation towards mesoderm and primitive endoderm [3] by repressing the neuroectodermal lineage fate [90]. Molecularly, this gatekeeper function is exerted by alternate partnering and changes in cobound factors to orchestra cell fate choice by alternating target gene binding.

#### 3.1.2. T-Box Transcription Factor 3 (Tbx3): Bystander in Pluripotency or Just Cell Fate Regulator?

Several studies have highlighted Tbx3 to function during mESC self-renewal [2–4, 65]. Briefly, the PI3K-AKT signalling pathway stimulates Tbx3 resulting in upregulated key pluripotency factor expression levels (Oct3/4, Nanog, and Sox2). To balance accurate pluripotency levels, Tbx3 expression is antagonised by the MAPK pathway [2]. It is also able to drive the expression of key pluripotency markers by direct promoter binding through Nanog [65]. In addition, Tbx3 acts as a downstream activator of WNT signalling [91]. WNT sustains the pluripotent state together with LIF, although, in the absence of LIF, WNT promotes cell differentiation towards primitive endoderm via Tbx3 [91, 92]. Hence, Tbx3 has been widely believed to belong to the inner core of the pluripotency circuitry, with loss of Tbx3 leading to differentiation. In marked contrast, we have recently identified fluctuating Tbx3 levels in mESCs: “Tbx3-low” cells resemble the gastrulating epiblast* in vivo* but retain the capacity to switch back to a Tbx3-high state. Moreover, we could show that Tbx3 is dispensable for the induction and maintenance of naïve pluripotency. Taken together, we delineate novel facets of Tbx3 action in pluripotency and show an involvement of Tbx3 in the transition from the naïve embryonic state to the prepatterned epiblast-like state. These purely mESC-derived data are further fostered by the observation of* in vivo* heterogeneity of Tbx3 in the ICM (Russell, Liebau, Kleger, unpublished data).

**Figure 3:**
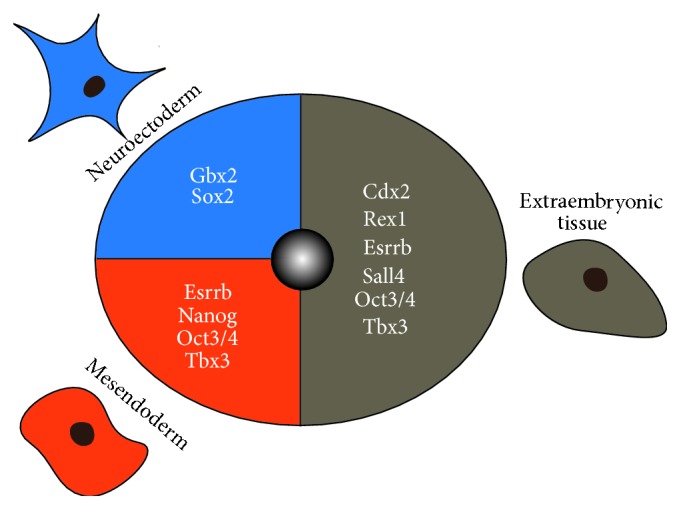
Spatial classifications of pluripotency factors. Upon exit of pluripotency core pluripotency factors get committed to distinct lineages and arising tissues, thus playing a crucial role in directing early lineage decisions in mouse. This image illustrates their spatial classification in the early embryo: neuroectoderm is mainly promoted by Gbx2 and Sox2 and mesendoderm especially by Esrrb, Nanog, Oct3/4, and Tbx3. Further, the extraembryonic tissue is induced by Cdx2, Rex1, Esrrb, Sall4, Oct3/4, and Tbx3. Cdx2: caudal type homeobox  2, Esrrb: estrogen-related receptor *β*, Gbx2: gastrulation brain homeobox  2, Tbx3: T-box transcription factor 3, Oct3/4: octamer binding transcription factor 3/4, Rex1: Zfp42, Sall4: Sal-like 4, and Sox2: SRY- (sex determining region Y-) box  2.

However, Tbx3 also fulfills a lineage specifying role, thus being classified as a mesendodermal-class gene [5] (Figure 3). We showed that Tbx3 is dynamically expressed during specification of the mesendoderm lineages in differentiating mESCs* in vitro* and in developing mouse and* Xenopus* embryos* in vivo* [4]. Nodal patterns the preimplantation embryo by interacting with the epiblast and the extraembryonic tissues [93]. Tbx3 overexpression promotes mesendodermal specification by activating crucial lineage specifying factors and by enhancing paracrine Nodal/SMAD2 signalling. Also, Tbx3 expression has also been detected in the visceral endoderm lineage. Therefore we suggest that Tbx3 may promote mesodermal lineage formation by activating Nodal via visceral endodermal cells expressing Tbx3 (Figure 2). Tbx3 is highly enriched in definitive endoderm progenitor cells [94] and it is involved in endoderm patterning together with chromatin-modifying enzymes [95]. The underlying mechanism includes spatial reorganisation of chromatin, leading to sensitisation for definitive endoderm promoting signals by enabling the histone demethylase Jmjd3 to transiently bind the two T-box factors: Tbx3 and Eomes.

In summary, Tbx3 is embedded in the core pluripotency networks to sustain stemness but also strongly directs early embryonic development by either transcriptional activation of differentiating gene programmes or modification of chromatin structures.

#### 3.1.3. Krüppel-Like Family (Klf): The Transcriptional Lineage Inhibitors

Klfs (Klf2, Klf4, and Klf5) are conserved zinc-finger-containing TFs implicated in various biological processes, such as proliferation, differentiation, and development [96]. In the early embryo they are already expressed in the ICM [97] (Figure 2). Klfs are indispensable for self-renewal by forming a tightly regulated molecular circuitry [64, 98]. Klf4 and Klf5 especially transcriptionally regulate expression of Nanog [98], a known inhibitor of differentiation during pluripotency [99, 100]. However, Klf2 is mainly regulated by Oct3/4, whereas Klf4 is regulated by LIF/STAT3 and additionally by Oct3/4. Klf5 is solely activated by LIF/STAT3 leading to ground state mESC pluripotency [101]. Previous reports suggest that LIF/STAT3 regulate Nanog and indirectly the core regulatory complex consisting of Nanog, Oct3/4, and Sox2 through activation of Klf4, Klf5, and Sall4 [29]. Despite their close relationship Klf4 and Klf5, they often exert opposite effects in regulating gene transcription and cellular proliferation* in vitro* and* in vivo* [102]. Klf4 is a negative regulator of mESC proliferation, whereas Klf5 acts as an activator. Also, Klf5 and Klf4 abrogate the mutual promoter effects [103]. In summary, the propagation of the LIF/STAT3 reinforced self-renewal state by Nanog is mediated by members of the Klf family. Their overexpression reinforces the pluripotent state in the absence of LIF [64, 104, 105], whereas their inactivation in mESCs induces spontaneous differentiation [29]. However, due to structural and functional overlaps, Klfs are able to compensate for each other. This was noticable as a triple knockdown abolished Nanog promoter activity and led to differentiation of mESCs [98], whereas single knockdowns did not exhibit a specific phenotype [98, 106].

However, Klfs do not just ensure the self-renewal state but are also critical components in regulating lineage specification in the early embryo. Recent work showed that Klf4 negatively regulates the visceral lineage (especially via GATA-binding factors 4 and 6 (GATA 4/6)) and definitive endoderm (especially via Sox17), whereas Klf5 primarily suppresses mesodermal differentiation (T, Mixl1 (mix paired-like homeobox  1)) (Figure 2) [107]. Thus, mESC pluripotency is ensured by additive inhibitory effects of Klf4 and Klf5. When we look at previous findings, Klf levels drop rapidly upon onset of lineage specification, resulting in a diminished lineage inhibition and subsequently in endodermal and mesodermal lineage formation [29]. Thus, mESCs are primed upon the drop of Klfs expression levels and prepared for early lineage differentiation.* In vivo*, Klf4-null mice survive early developmental stages but die shortly after birth due to tissue abnormalities within the smooth and cardiac muscle and basal membrane formation problems which primarily is due to a defective GATA4 regulation [108, 109]. A lacking gastrulation phenotype in Klf4-deficient mouse embryos suggests compensatory mechanisms by the Klf family. In contrast, Klf5-deficient embryos fail to develop further than the blastocyst stage due to reduced Oct3/4 and Nanog expression levels [64, 110]. Taken together, Klfs exert a great impact in sustaining the self-renewal state by either interacting with core pluripotency activating genes or inhibiting differentiation. However, the inhibition of endodermal structures primarily by Klf4 and mesodermal structures by its counterpart Klf5 does not just safeguard the self-renewal state but balances accurate differentiation onset in the early mouse embryo and in differentiating PSCs (Figure 2).

#### 3.1.4. Nanog: Various Facets during Differentiation and Self-Renewal

Nanog, the homeodomain TF, acts together with Oct3/4 and Sox2 to maintain the pluripotency network through the Oct/Sox-motif [74, 98, 111, 112]. Nanog is also able to sustain self-renewal independently from the LIF/STAT3 pathway [113], albeit at a reduced self-renewal capacity [74]. Most robust self-renewal state is promoted under continuous Nanog overexpression and LIF stimulation [114]. However, while Nanog has previously been shown to be dispensable for mESC culture, it mainly functions in stabilising the pluripotency network [115]. Previous studies hypothesised that a switch from monoallelic Nanog expression, in a LIF/serum setting, to a biallelic manner in 2i conditions results in higher expression levels. Of note, this hypothesis was disproved by recent results illustrating a steady biallelic expression of Nanog [116, 117]. Despite direct transcriptional regulation of pluripotency target genes, Nanog sustains the self-renewal state by inhibiting several differential processes in mESCs. First, a population of early mesoderm-specified progenitors was identified, normally present in mESCs and promoted by the BMP pathway. These primed mESCs express pluripotency-associated TFs such as Oct3/4 and Rex1 but are actually specified to the mesodermal fate. In the presence of LIF, Nanog is able to respecify mesoderm-specified progenitors back to pluripotent mESCs [118]. A second mechanism of blocking the progression of mesoderm is a direct inhibition of SMAD1 via STAT3 activation in a LIF-containing setting [119], which has also been demonstrated for Oct3/4 [120], suggesting their cooperative function to sustain self-renewal.

Similarly, Nanog functions as an important determinant during cell fate decisions at the blastocyst stage* in vivo* [121]. Here, it is downregulated by Cdx2 in the trophectoderm [85], whereas it is highly expressed in the ICM [122, 123]. The early ICM of the blastocyst gives rise to the epiblast and primitive endoderm progenitors as a first sign of lineage choice occurring in the ICM. In the unrestricted, early ICM of the blastocyst, Nanog expression is dependent on transcriptional binding of Oct3/4, Sox2, and Esrrb [112, 124], while, in the later derived epiblast, Nanog promoter binding changes now being dependent on Activin A signalling via SMAD2 [123, 125, 126]. We know that Nanog is essential for both epiblast and primitive endoderm formation (Figure 2). GATA6 is crucial in the primitive endoderm lineage* in vivo* and* in vitro* [113, 127–129] and is transcriptionally repressed by Nanog in epiblast-engaged cells [130]. However, recent studies showed primitive endoderm to require epiblast cells for proper differentiation, whereby Nanog signals through FGF/ERK signalling (FGF4) resulting in upregulated GATA4 and Sox17 expression levels [121]. This observation has been recently fostered by live-time imaging data using a Nanog-reporter system* in vivo*, where Nanog expression marks an irreversible commitment between the epiblast and the primitive endodermal lineage, while rarely unidirectional conversions from primitive endoderm to epiblast occur [131].

Elegant studies from the Vallier Laboratory combined PSCs and developing mouse embryos to uncover the transcriptional network around Nanog during pluripotency and lineage commitment [132]. Herein, SMAD2/3 controls the self-renewal state by interacting with core TFs, such as Oct3/4 and Nanog. Notably, endodermal differential programmes were also shown to be downstream of SMAD2/3, demonstrating an opposing function of SMAD2/3. As SMAD2/3 and Nanog are involved in both processes, SMAD2/3 may be guided by distinct genes like Nanog to achieve tissue specific functions [133]. Further, during primitive streak formation initial pluripotent differentiation inhibitors rapidly decline beginning with Sox2. This enables Nanog to form complexes with SMAD2/3, which promote Eomes expression levels. Eomes further diminishes pluripotent signals thereby ensuring sufficient Nanog expression. Subsequently, Nanog expression levels decline making room for SMAD2/3 and Eomes thereby directing primitive streak cells towards endodermal fate and coincidently inhibiting mesoderm formation [134]. This observation receives further support from another recent study showing that Nanog cooperates with Activin/SMAD in recruiting histone modifiers such as Dpy30, a subunit of the COMPASS methyltransferase complex, thus regulating differentiation-linked genes [132].

In line with these data, Nanog has been classified as a mesendodermal-class gene, like Oct3/4 and Tbx3 [5] (Figure 3). All these genes reveal overlapping gene expression levels and lineage specifying patterns, albeit we are still lacking evidence demonstrating their mutual relationship during early lineage decisions [5, 74]. Inconsistent data has been published regarding the Nanog-lacking phenotype. Initially, Nanog deficiency was reported to result in a failure of epiblast formation and, concomitantly, mESCs lost their pluripotent fate and differentiated into trophectoderm [113]. However, this phenotype was not clearly replicable in recent studies [130]. Instead, Nanog-deficient embryos reveal upregulated GATA6 expression levels and few GATA4-positive cells [130]. Therefore, Nanog mutant ICMs do not undergo apoptosis suggesting a stabilising role of GATA6 in this regard [130]. In summary, Nanog shows multiple facets in regulating mESC pluripotency or by specifying lineage formation in the early mouse embryo.

### 3.2. Neuroectoderm-Class Genes

#### 3.2.1. Sex Determining Region Y- (SRY-) Box  2 (Sox2): The Neuroectodermal Embryonic Stem Cell Gene

Another master regulator of mESC pluripotency is Sox2, a member of the HMG (high-mobility group) box proteins. Sox2 maintains stemness by directly interacting with Oct3/4 in a reciprocal fashion. Both share the ability to bind to a unique promoter region, the Oct/Sox-motif [135]. This highly conserved Oct/Sox-element is critical for transcriptional regulation of pluripotency and located on different genes in undifferentiated mESCs, such as Oct3/4, Sox2, and Nanog [112]. The proper modulation of these target genes preserves the self-renewal state [88]. Notably, Niwas group showed that Sox2 was dispensable for activating the Oct/Sox-element [88]. Instead, Sox2 is important in activating pluripotency-associated genes, which in turn regulate Oct3/4 resulting in stable Oct3/4 expression levels. Both activate FGF4 expression in the ICM and epiblast [136] and in turn FGF4 controls ICM maintenance and subsequently interacts with Cdx2 and Eomes while promoting trophectoderm maturation [85, 137].

During early lineage decisions, Sox2 is broadly expressed in the ICM and the trophectoderm and later even in the epiblast and in the primitive endoderm [138, 139] (Figure 2). Interestingly, Sox2 knockout studies indicated stable or even low Oct3/4 and Nanog expression levels and at the same time surprisingly low trophectoderm gene (Eomes, FGF4) expression levels [139], resulting in embryonic lethality due to defective epiblast development during the peri-implantation stage. Stable Nanog and Oct3/4 levels seem unusual, as Sox2 is a central member of the pluripotency-maintaining protein complex. Nevertheless, this effect could be explained by autoregulatory functions, which Sox2 has in common with Oct3/4, Nanog, Sall4, and Klfs [98].* In vitro*, upon reduction of Sox2 levels, mESCs differentiate towards trophectoderm indicating its impact in sustaining the pluripotent state. Thus, trophectoderm differentiation could result from a secondary loss of Oct3/4 levels due to Sox2 reduction [88]. Heterozygous Sox2 knockout mutations in mouse show severe alterations in brain and neural cells [140], thus gaining insight into the promoted germ layer. Sox2 induces neuroectodermal gene expression [5] by specifically repressing Oct3/4, which vice versa participates in cell fate choice by promoting the mesendodermal lineage [86] (Figure 2). In summary, Sox2 is embedded in the pluripotency network by interacting with key pluripotent genes. However, upon onset of differentiation, Sox2 promotes neuroectodermal lineage allocation by transcriptional repression of Oct3/4 (Figure 3).

#### 3.2.2. Gastrulation Brain Homeobox  2 (Gbx2): Another Ectodermal Player?

The TF Gbx2 is a direct downstream target of LIF/STAT3. Its overexpression is able to substitute for LIF in mESCs and to maintain self-renewal in a STAT3 deprived mESC culture [141]. Notably, Gbx2 is able to push primed EpiSCs back into the pluripotent state [141]. Nonetheless, its impact on pluripotency just seems to have a supporting character, as shown by* in vitro* knockdown studies, where the pluripotent state was not fully impaired [141]. This is in line with* in vivo* studies, which do not show morphological abnormalities in the blastocyst [141]. In the mouse embryo, Gbx2 is expressed in the ICM and, together with Rex1 (Zfp42), diminishes during primitive ectoderm formation [142] (Figure 2). Notably, at gastrulation stage, Gbx2 is present in all three germ layers [143] but subsequently gets upregulated in the neural ectoderm and underlying mesoderm and finally is limited to the CNS (central nervous system) [144] (Figure 2). In line, Gbx2 knockout mice show abnormalities in hindbrain development, neural crest patterning, and cardiovascular and craniofacial defects and die soon after birth [143, 145].

Taken together, although Gbx2 lies downstream of LIF/STAT3, it seems to be dispensable for mESC culture and ICM integrity. However, during early developmental steps, Gbx2 is committed to the ectodermal lineage fate (Figure 3) [146].

### 3.3. Extraembryonic-Class Genes

#### 3.3.1. Spalt-Like Transcription Factor 4 (Sall4)

The transcriptional network in preimplantation embryos was recently extended by the spalt-like gene family member Sall4. Sall4 directly interacts with Nanog and cooccupies several gene sites, including their own promoters, forming autoregulatory loops. Together, they are suggested to function as a Sall4/Nanog-complex maintaining pluripotency by reciprocal regulation. The physical relationship was suggested to be similar to the Oct3/4-Sox2 interaction encountered on many loci in mESCs [147]. Besides, in the ICM and the epiblast, Sall4 expression occurs simultaneously to Oct3/4 and Sox2 expression [148], revealing another contribution to the transcriptional network. This was confirmed by verifying Sall4 occupation on the Oct/Sox-element* in vitro* and* in vivo* [149] and by gene profiling analyses illustrating Sall4 cooccupying the same target genes as Sox2 in mESCs [149]. Independently, Sall4 can modulate Oct3/4 expression levels, the master TF of pluripotency, and vice versa, indicating its critical role in maintaining stemness [150]. In summary, Sall4 contributes to the transcriptional network by direct interaction with the key players, Nanog, Oct3/4, and Sox2, to maintain the self-renewal state. Moreover, Sall4 can also be recruited to the promoters of Klf2 and Klf5, although its role contributing to mESC pluripotency through the Klf family requires further studying. Another possible mechanism supporting Sall4 mediated pluripotency could be the recruitment of the strong transcriptional repressor complex NuRD (nucleosome remodelling and deacetylase) [151]. Both Sall4 and NuRD are expressed in mESCs and are involved in maintaining stemness. As Sall4 associates with the NuRD complex, both genes could act together in sustaining pluripotency. But due to lacking evidence this hypothesis remains an assumption.

At the blastocyst stage, Sall4 regulates the ICM development to the two blastocyst-derived stem cell lineages: epiblast and extraembryonic endoderm (Figure 2). GATA6 is important in defining the extraembryonic endoderm, although GATA6 mutants exhibit lineage specific defects, a short time after blastocyst formation. Thus, GATA6 does not seem to be a core component for primitive endoderm initiation but rather for its maturation [152]. In this regard, studies exhibited Sall4 to regulate extraembryonic endoderm genes, such as GATA4, GATA6, Sox7, and Sox17 [153]. So it can be concluded that Sall4 contributes to the transcriptional network maintaining mESC pluripotency mediated by direct interaction with crucial TFs [154]. In line with this, it is not surprising that Sall4 overexpression leads to primitive endoderm differentiation [154] and further may support Oct3/4 in this matter [87]. Also, similar to Oct3/4, Sall4 knockdown mESCs are becoming alkaline phosphatase (AP) negative and tend to differentiate towards the trophoblast lineage [148, 154]. It is feasible that Sall4 cooperates with Oct3/4 to suppress trophectoderm lineage allocation [155]. Sall4-null mice are lethal during peri-implantation [156, 157], whereas heterozygous mice exhibit anorectal tract abnormalities, heart defects, skeletal defects, and anencephaly [157].

Taken together, Sall4 is broadly connected within the pluripotency network and has a major role in driving early lineage decisions especially towards the extraembryonic lineage (Figure 3).

#### 3.3.2. Estrogen-Related Receptor *β* (Esrrb)

The estrogen-related receptor beta (Esrrb), a member of the nuclear orphan receptor family, is a core pluripotency member of sustaining naïve pluripotency and is deeply embedded in the self-renewal network interacting with several pivotal TFs [158]. It transcriptionally interacts with Nanog in a reciprocal manner [51] and apart from overlapping target gene profiles. Esrrb is able to substitute for Nanog in mESCs [159]. Further, Esrrb binds promoters of the master pluripotency factor Oct3/4 [160] and other TFs such as Klf2 [161] and Rex1 [160]. It acts independently of LIF/STAT3 [51]. Despite its expression levels being directly modulated by the transcriptional repressor Tcf3, Esrrb mediates self-renewal effects upon GSK3 inhibition and is also able to stabilise *β*-catenin levels. Thus, in the presence of a MEK inhibitor, for example, by the small molecule PD03, Esrrb overexpression can replace GSK3 inhibition maintaining self-renewal in mESCs [51, 159]. Indeed, mESCs depleted from Esrrb and cultured in the absence of LIF undergo morphological changes, reduction of AP activity resulting in spontaneous differentiation [51, 162].* In vivo*, Esrrb deletion leads to embryonic lethality during midgestation due to placenta defects [163]. This is in line with previous studies, which indicated the presence of Esrrb in the trophoblast lineage [164]. Surprisingly these embryos survive throughout gastrulation exhibiting no defects in the ICM and epiblast [163]. Thus, we presume that Esrrb can be compensated by other factors in this complex transcriptional network maintaining pluripotency.

Regarding its expression profile in the ICM and trophoblast (Figure 2) and the fact that Esrrb enhances Cdx2 expression levels, it is also fair to assume that Esrrb acts as an important factor in regulating lineage decisions in the early blastocyst. As we know, overexpressed pluripotency factors promote differentiation. This was also shown for Esrrb, as overexpressed mESCs are prone to differentiate towards the endodermal lineage [165, 166]. Further investigations are necessary to clarify molecular mechanisms to date, but regarding its attested role in trophoblast specification we classified Esrrb to be an extraembryonic-class gene (Figure 3).

#### 3.3.3. Rex1 (Zfp42)

Several studies have successfully used all-trans retinoic acid (RA) to induce mESC differentiation [167]. The stem cell marker Rex1 reduces RA-induced differentiation thereby sustaining the self-renewal state [168–170]. During mESC pluripotency, Rex1 activity is up- or downregulated by Oct3/4 depending on its expression levels [171]. Also Sox2, another key pluripotency factor, is able to transactivate Rex1 via Nanog [169]. Nonetheless, Rex1 is dispensable for mESC pluripotency [172].

Endogenously, Rex1 is expressed in the ICM and in trophoblast lineages [173] (Figure 2). Notably, unlike Oct3/4, Rex1 is not present in all cells of the ICM. A previous study illustrated two different subpopulations: both were positive for Oct3/4 but only one of them was Rex1-positive [174]. Notably, they were able to convert into each other upon LIF stimulation. The group double positive (Rex1^+^/Oct3/4^+^) developed into primitive ectoderm and participated in chimera formation, whereas the other group, Rex1^−^/Oct3/4^+^, induced somatic differentiation, indicating the existence of primed subcultures in ICM which are guided by Rex1 to develop into either epiblast cells or primitive endoderm [174].

Overexpression of Rex1 in mESCs surprisingly results in impaired self-renewal [175] and* in vivo* delayed development through early cleavage divisions [176]. However, homozygous Rex1 knockout in mESCs causes spontaneous differentiation into all three primary germ layers [168, 170, 177], indicating that Rex1 can at least reduce RA associated differentiation. To date, there is neither a noticeable phenotype regarding Rex1-deficient mESCs nor mice.* In vivo* experiments displayed Rex1-deficient mice to be fertile and viable but their offspring die at late gestation [172]. All in all, as the pluripotency factor Rex1 seems to drive lineage segregation at the early blastocyst stage especially towards the extraembryonic lineage, we decided to position it in this section (Figure 3).

### 3.4. Epigenetic Modulations

*Methyl-Binding Domain Protein 3 (Mbd3)*. Epigenetically, transcriptional sustainment of mESC pluripotency is modulated by chromatin remodelling including histone modifications and DNA methylation. Pluripotency is maintained by a balance between LIF/STAT3 activation and a repression through NuRD [178]. NuRD mediated silencing in mESCs plays a critical role in early lineage commitment into all three germ layers [179]. Mbd3 is the key subunit of the NuRD repressor complex network [180]. A recent publication demonstrated Mbd3 to be dispensable for maintaining mESC pluripotency. Also, its expression seems to diminish upon fertilisation, therefore being absent during the early preimplantation period, and is upregulated towards the late morula/blastocyst stage [181]. However, according to previous work, Mbd3 exhibits a critical role repressing the trophectoderm lineage in order to ensure mESC propagation and subsequently proper differentiation of the epiblast [182]. Additionally, NuRD mediated mESC self-renewal was shown to function through epigenetic modulation of the WNT pathway [183].

As epigenetic modifications occur during ICM and primary germ layer formation, it is not surprising that Mbd3 deletion in mice leads to early embryonic lethality [184], thus being indispensable for early embryonic development.

## 4. Conclusion

Naïve pluripotency implies the ability of unlimited self-renewal and regeneration. Its complex regulatory circuity has been studied extensively. Key players such as Oct3/4, Nanog, and Sox2 are complemented by many TFs forming a tightly regulated network that sustains the self-renewal state by inhibiting genes required for differentiation. Upon withdrawal of stemness-inducing signals, pluripotency factor expression levels decline, giving differentiation factors the opportunity to induce lineage allocation. These cell fate decisions are essential for developmental processes into the primary germ layers.

Interestingly, several members of these pluripotency-associated factors have been identified to exert dual functions during early embryogenesis, by also governing the transition of pluripotency towards early lineage commitment. Here, TFs initially maintaining pluripotency by activating self-renewal gene programmes undergo a molecular switch resulting in enhanced expression levels of differentiation inducing genes. In this regard, the TFs were classified into mesendoderm-, neuroectoderm-, and extraembryonic-class genes (Figure 3). Notably, mesendoderm-class genes inhibit neuroectoderm and vice versa to promote the appropriate lineage. Although both the pluripotency circuitry and early lineage commitment mechanisms have been studied in great detail, there is still a broad gap of knowledge regarding the distinct regulatory complexes governing these events. By understanding the interplay of factors and signalling pathways involved in the early embryonic development, basic as well as clinical science would be able to profit broadly in terms of developmental steps and pathomechanistical events. Summarising, TFs interact in network in a spatial and temporal manner to exit pluripotency and establish different lineages in the early embryo.
